# Vocal fingerprinting reveals a substantially smaller global population of the Critically Endangered cao vit gibbon (*Nomascus nasutus*) than previously thought

**DOI:** 10.1038/s41598-023-50838-2

**Published:** 2024-01-03

**Authors:** Oliver R. Wearn, Hoang Trinh-Dinh, Chang-Yong Ma, Quyet Khac Le, Phuong Nguyen, Tuan Van Hoang, Chuyen Van Luong, Tru Van Hua, Quan Van Hoang, Peng-Fei Fan, Tho Duc Nguyen

**Affiliations:** 1 Fauna & Flora, Vietnam Programme, Hanoi, Vietnam; 2 School of Bioresources and Technology, King Mongkut’s University of Technology Thonburi, Bangkok, Thailand; 3 College of Life Sciences, Guangxi Normal University, Guilin, China; 4 Trung Khanh Ranger Station, Forest Protection Department, Ministry of Agriculture and Rural Development, Trung Khanh, Cao Bang Vietnam; 5 School of Life Sciences, Sun Yat-sen University, Guangzhou, Guangdong China

**Keywords:** Conservation biology, Tropical ecology, Zoology

## Abstract

The cao vit gibbon (*Nomascus nasutus*) is one of the rarest primates on Earth and now only survives in a single forest patch of less than 5000 ha on the Vietnam–China border. Accurate monitoring of the last remaining population is critical to inform ongoing conservation interventions and track conservation success over time. However, traditional methods for monitoring gibbons, involving triangulation of groups from their songs, are inherently subjective and likely subject to considerable measurement errors. To overcome this, we aimed to use ‘vocal fingerprinting’ to distinguish the different singing males in the population. During the 2021 population survey, we complemented the traditional observations made by survey teams with a concurrent passive acoustic monitoring array. Counts of gibbon group sizes were also assisted with a UAV-mounted thermal camera. After identifying eight family groups in the acoustic data and incorporating long-term data, we estimate that the population was comprised of 74 individuals in 11 family groups, which is 38% smaller than previously thought. We have no evidence that the population has declined—indeed it appears to be growing, with new groups having formed in recent years—and the difference is instead due to double-counting of groups in previous surveys employing the triangulation method. Indeed, using spatially explicit capture-recapture modelling, we uncovered substantial measurement error in the bearings and distances from field teams. We also applied semi- and fully-automatic approaches to clustering the male calls into groups, finding no evidence that we had missed any males with the manual approach. Given the very small size of the population, conservation actions are now even more urgent, in particular habitat restoration to allow the population to expand. Our new population estimate now serves as a more robust basis for informing management actions and tracking conservation success over time.

The Critically Endangered cao vit gibbon (*Nomascus nasutus*) is one of the rarest primates on Earth, with only a single, small population remaining on the Vietnam-China border. It now occurs in just one forest block totalling 4839 ha, not all of which is suitable habitat for the gibbon. Conservation measures have been in place for the species since it was rediscovered in 2002 in Vietnam^1^ and re-confirmed in China in 2006^2^, including: the establishment of two protected areas; regular patrolling by rangers and community groups; habitat restoration; support for sustainable livelihoods; awareness-raising about the plight of the gibbon, and educational events with local schools^3^.

Alongside these activities, periodic surveys of the cao vit gibbon population have been done, to inform management decisions. Specifically, surveys have provided data for population viability analyses, informed prioritisation of different conservation interventions, and helped to track the impact of interventions over time. Surveys to date have estimated a population size of around 120 individuals, with the lowest and highest estimate of 109 (in 2018) and 137 (in 2012), respectively^3,4^. However, for a long time, surveyors have been aware of substantial subjectivity inherent in population survey methods for gibbons^5^. Most gibbon surveys to date, including all those done for the cao vit gibbon, have estimated density or abundance by triangulating group locations from multiple survey posts that are monitored simultaneously^6,7^. Gibbons are sometimes observed directly but, most often, are detected indirectly from their songs. Crucially, the triangulation method depends on being able to reliably match gibbon groups across different surveyor teams, based on reported bearings and distances from surveyors, and the recorded start and end times of any songs. Surveys typically occur over multiple days (to ensure no gibbon groups are missed) and so gibbon groups must also be matched successfully from one day to the next^6,7^. If two groups sing close together at different times, there is a risk that they are identified as one group and the total population size is under-estimated. Equally, if a single group moves quickly to a new location and sings again, or moves a far distance between survey days, there is a risk that the group is identified erroneously as two groups, and the total population size is over-estimated. In addition, distances and bearings are difficult to estimate in the field and are likely associated with substantial (and unquantified) error. This is especially likely to be the case in the complex topography of the cao vit gibbon’s karst habitat. Due to these factors, we suspect previous cao vit gibbon population estimates have suffered from bias.

For the cao vit gibbon, an indicator of this bias has long existed: a discrepancy between the population survey data from Vietnam and China. In China, researchers have been intensively following gibbon groups since 2007^8,9^ and have a detailed understanding of their home ranges; this was not the case in Vietnam until focal group monitoring began finally in 2020^3^. The monitoring data from China revealed that exclusive home ranges (excluding overlaps) were approximately 100 ha^10^ and density was 1.0 groups per km^2^ (five groups occupying 4.9 km^2^). However, inferred home ranges and densities from Vietnam-only groups are 26 ha and 3.8 groups per km^2^, respectively (16 groups occupying 4.2 km^2^; based on 2018 population survey data^11^). Unless the resource availability of the occupied area on the Vietnam side of the border is substantially higher (for which there is no clear evidence^3,12^), these numbers suggest an over-estimation of the population in Vietnam.

A key characteristic of the family Hylobatidae is their singing behaviour. Like in other gibbon species, cao vit gibbon family groups sing during most mornings, with the male providing four main phrase types—‘staccato’, ‘boom’, ‘multi-modulated’ and ‘coda’—and the female contributing the ‘great call’ (Fig. 1)^13^. In multi-female groups, which are the norm in the cao vit gibbon (all of the studied groups in China had two adult females^14,15^), the females will synchronise their great calls. Community monitoring teams in Vietnam, as well as research teams in China, have long reported that individual gibbon groups have distinctive songs. Gibbon species within the genus *Hylobates* have also long been known to show individuality in their songs, in particular in the female great call, and gibbons may themselves use this information to decide whether to escalate conflicts^16,17^. This has been studied far less in the genus *Nomascus*, but work on the western black crested gibbon (*N. concolor*) and indeed the cao vit gibbon has shown that songs, in particular the male songs, are highly individualistic and stable over time^17–19^. This has led to the suggestion that these vocal ‘fingerprints’ in gibbons could be used as the basis of an objective population survey method^19,20^. To our knowledge, a population survey using this approach has never been done for a gibbon species. This is likely because previous acoustic studies of gibbons have employed a manual recording approach, using high quality and expensive directional microphones^16–21^, which is resource-intensive and difficult to carry out at scale.

**Figure 1:**
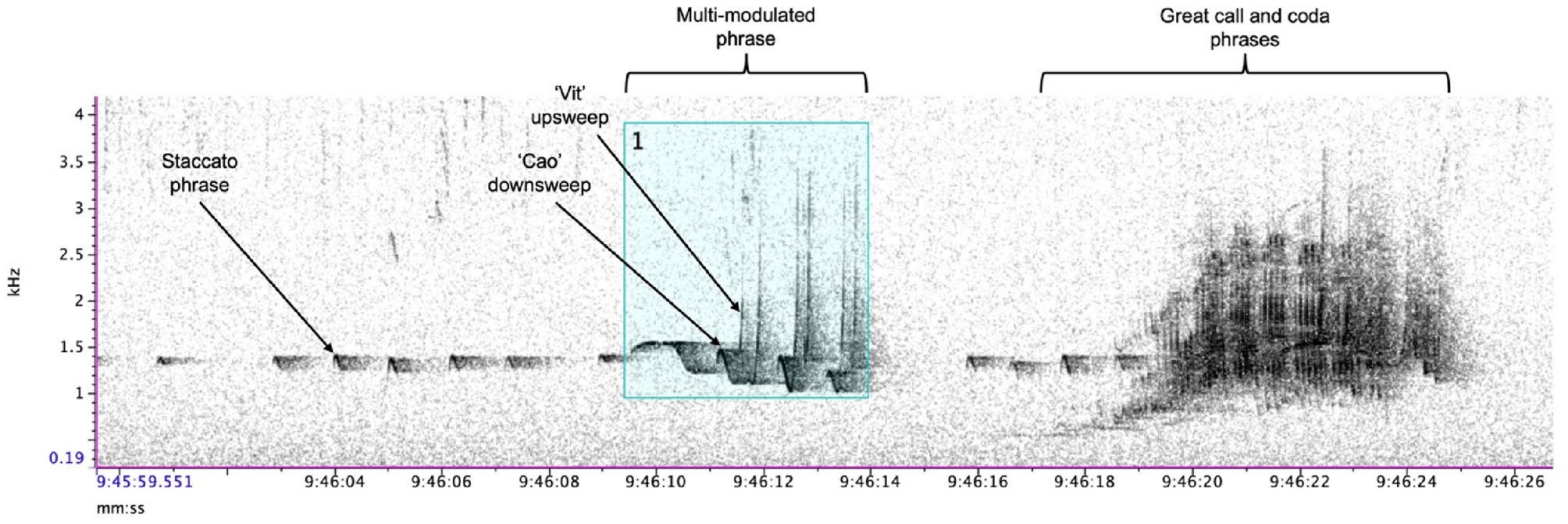
Example spectrogram of a cao vit gibbon duet, showing the male (staccato, multi-modulated and coda) and female (great call) contributions. Males also have a boom phrase, not shown here. Audio was recorded using an AudioMoth and the spectrogram was visualised using Raven Pro (with settings as given in the main text). An example bounding box annotation around the male multi-modulated phrase is also shown (in blue).

Here we report on a transboundary survey of the entire cao vit gibbon population, for the first time complementing traditional observational data from survey teams with acoustic data from passive acoustic monitoring. We used low-cost ‘AudioMoth’ devices for the acoustic monitoring^22^, allowing us to deploy devices at scale. The observations of survey teams were also supplemented with data from an unoccupied aerial vehicle (UAV) equipped with a thermal camera. The UAV proved important for determining accurate group size counts, which we have reported on previously^23^. We find that this multi-method approach, including vocal fingerprinting analysis, offers a promising new approach for gibbon surveys and we are able to set a new, robust population baseline for the cao vit gibbon.

## Materials and methods

### Study sites

The population survey was spread across two adjoining protected areas: the Cao Vit Gibbon Species and Habitat Conservation Area (SHCA) in Trung Khanh District, Cao Bang Province, Vietnam and the Bangliang National Nature Reserve (NNR) in Jingxi County, Guangxi Autonomous Region, China. Habitat in this area is composed of subtropical monsoonal forest, growing on a complex karst substrate with densely-packed hills and deep valleys (elevation range 500–930 m). Habitat on both sides of the border was historically subject to intense pressures from timber extraction, charcoal production, firewood collection, grazing and hunting, with some valley areas clear-cut for agriculture^3,12^. Threats have declined substantially over time, however, with the forest now in a state of recovery^3,12^. We hypothesise that this is due to the reduced forest-dependence of local livelihoods over time, and the demarcation and management of protected areas for the gibbon (since 2007 and 2009 for the Cao Vit Gibbon SHCA and Bangliang NNR, respectively).

### Observational survey

**Figure 2:**
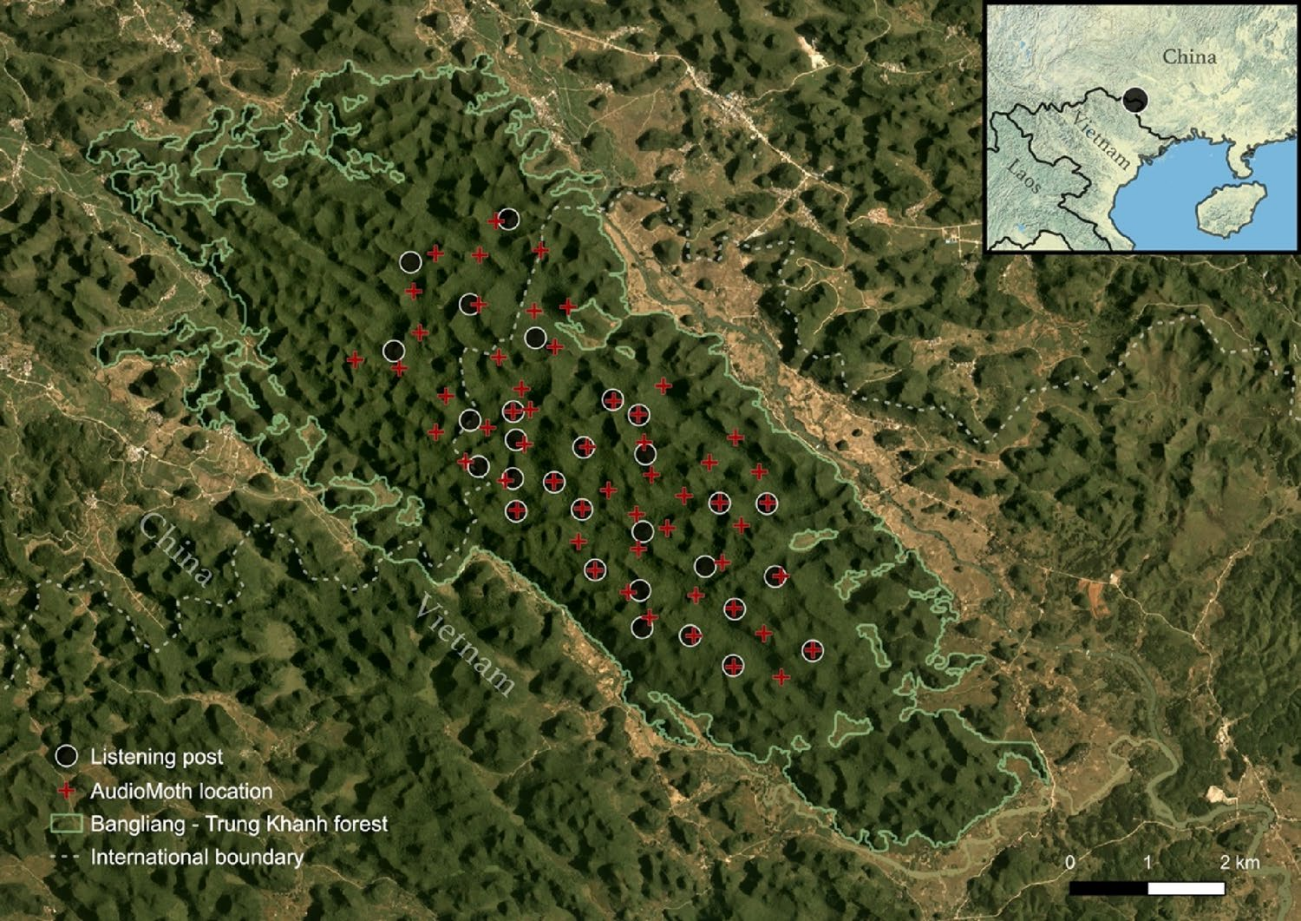
Survey design for listening posts and AudioMoth recorders used in this study. The global population of the cao vit gibbon is restricted to the Bangliang—Trung Khanh forest block (green outline). The sections of this forest in China and Vietnam are protected within the Bangliang National Nature Reserve and Cao Vit Gibbon Species and Habitat Conservation Area, respectively (boundaries not shown). Basemap is a PlanetScope image from 2020. Inset map shows the location of the study site within east Asia.

A total of 29 listening posts were distributed across the global range of the cao vit gibbon (23 in Vietnam, four in China and two along the border), including all areas known to be occupied on the basis of long-term monitoring, as well as areas we thought could conceivably hold newly-formed groups (Fig. 2). Listening posts were separated by a mean distance of 689 m (range 367–1206 m), leaving no gaps within which singing gibbons could be missed (given that songs can often be heard at distances > 1 km). Listening posts were placed on mountain tops and ridges to maximise the probability of hearing and observing groups. We included in the design almost all listening posts used in previous surveys, removing five which were deemed to be duplicates of other posts, and included eight additional listening posts to cover areas of potential expansion of the population.

We planned for each listening post to be monitored for at least five consecutive mornings, which we deemed to be sufficient to detect all groups. Given a daily singing probability (*p*_daily_) of 0.7^24^, the probability of detecting a group at least once (*P*) over five mornings is: *P*_*5*_ = 1 – (1 – *p*_daily_)^5^ = 1 – (1 – 0.7)^5^ = 1.0. We anticipated that bad weather might reduce our actual survey effort to three mornings, in which case *P*_*3*_ = 0.97, which we still deemed satisfactory.

For comparability with previous surveys, we undertook the survey after the peak of the rainy season (i.e., in the period September to November), a time which offers favourable field conditions and when, anecdotally, we knew that cao vit gibbons have sung frequently in previous years. Due to the availability of human resources, we split the survey into two blocks, with 13 and 19 listening posts monitored in the first and second phases, respectively, of which three listening posts in the middle of the survey grid were maintained over the whole period to ensure groups in the area of overlap were not double-counted over the two periods.

On each survey day, teams of two to three surveyors monitored listening posts from approximately one hour before dawn until at least four hours after dawn (05:00–10:00 in October), covering almost the entire time that cao vit gibbons are known to sing^24^. Logistics-permitting, teams extended the observation time until 12:00 (in Vietnam) or 17:00 (in China). If gibbons were heard or sighted, the start and end times of the detection, group composition, bearing and distance were recorded. Song bouts were considered independent if separated by more than five minutes or if clearly from a different group. To help field teams validate their bearing and distance measurements, locations were also plotted over contour maps in the field, either on paper or in a custom-designed project built on the smartphone application Mergin Maps (Lutra Consulting Ltd., UK), depending on the capacity of field teams. At the end of each survey day, teams attempted to match gibbon detections based on the information recorded. At the conclusion of field work, the data from Vietnam were revisited, by discussing each survey day one-by-one and agreeing on the matched detections. One of us (TDH) led this process, with input from all surveyors. A further meeting held online (owing to Covid-19 movement restrictions) was held between teams in Vietnam and China to match the detections across both countries. These preliminary determinations were investigated further using the acoustic data (see below) and refined accordingly.

A roving team of two people also assisted the counting of group sizes using a UAV (DJI Mavic 2 Enterprise Advanced)^23^. This team operated independently of the main survey teams, each morning selecting whichever listening post was deemed to offer the best chance of detecting gibbon groups. For any detected groups, simultaneous thermal and RGB video was recorded (weather-permitting) at a distance of approximately 100 m. Videos were later reviewed carefully and group size and composition enumerated^23^.

### Passive acoustic survey

We also deployed AudioMoth acoustic recorders across the global range of the cao vit gibbon. A total of 55 locations were monitored, separated by an average of 501 m (range 224–737 m), covering all known and potential areas occupied by the cao vit gibbon (Fig. 2). Of these, 41 were deployed on mountain tops and ridges, mostly nearby to listening posts also used by survey teams. These were supplemented by 14 additional sites in valley-bottoms, which were logistically easier to access and, we hypothesised, might provide more precise localisation of gibbon songs given the more constrained area of detection in valleys. Preliminary testing of the AudioMoth’s ability to detect cao vit gibbons undertaken between September 2020 and February 2021 revealed that detection probability was approximately 0.9 at 500 m, declining to 0.5 at 1000 m (P. Nguyen, 2021, unpublished data); this means that we are very unlikely to have missed any groups within the survey grid. The survey was split into two phases: the first phase (18th October to 7th December 2021) was concurrent with the traditional observational survey and devices (n = 42) were only deployed in Vietnam (owing to Covid-19 restrictions on sending equipment to China), whilst in the second phase (7th December 2021 to 25th January 2022) devices were deployed across the entire area (n = 54). Here, we only use data from the first phase, for which we have overlapping field-based observations.

AudioMoth devices were programmed to record continuously every day from 04:30 to 12:00 at a sample rate of 16 kHz (recording one-hour files with five seconds between recordings). Devices were deployed inside waterproof (Ingress Protection X7) cases, fixed securely onto the end of 2 m high metal poles, and placed in micro-sites without obvious sound barriers nearby (such as dense vegetation or boulders). Devices were oriented in the direction that field teams deemed would be more likely to detect gibbons; in any case, preliminary testing found that detection probabilities were largely unaffected regardless of whether AudioMoths were facing towards singing gibbons or not (P. Nguyen, 2021, unpublished data).

### Spatially explicit capture-recapture (SECR) analysis

We input the distance and bearing measurements made by field teams into SECR analysis^25^. This allowed us to: (i) estimate the location of each calling group by reconciling the bearing and distance measurements from multiple teams; (ii) evaluate errors in the estimation of distances and bearings, (iii) characterise the detection function, which describes how detection probability declines as a function of distance, and (iv) identify drivers of variation in song density (i.e., songs per hour of survey per km^2^). We aimed to feed the results into the design of ongoing long-term monitoring, as well as the design of future surveys. By modelling song density, we also aimed to improve our understanding of the gibbon’s ecology and identify potential areas for protection and-or restoration. SECR can also be an effective method for estimating gibbon group density when surveys cover an unknown proportion of groups inhabiting an area. We did not exploit this here, given that we were able to survey the entire cao vit gibbon population.

Using the R package ‘ascr’ v. 2.2.4^26^, we ran two competing models for song density, each representing a distinct hypothesis about the factors determining where gibbons sing from. The *forest structure* model contained the variables tree canopy cover^27^ and canopy height^28^, whilst the *accessibility* model contained the variables distance from forest edge and elevation^29^. A quadratic term for elevation was also included in the accessibility model, to allow for non-linear responses to this covariate. In both models, we also included a binary variable for whether it rained or not during the survey day. We selected the best model on the basis of Akaike’s Information Criterion corrected for small sample size (AIC_c_). We used a half-normal detection function with the intercept parameter (g_0_) fixed at one; preliminary modelling with no covariates indicated that this was clearly preferred over a hazard detection function (ΔAIC_c_ =  − 21.5). Models were integrated over a grid of points overlaying the Trung Khanh—Bangliang forest block with 100 m spacing. Estimated locations for each gibbon song were output from the best model and, for each group with more than five locations, 95% kernel home ranges were calculated using the R package ‘ctmm’ v. 1.1.0 with default settings^30^. We consider these kernel home range estimates as indicative only, given the small sample sizes.

### Vocal fingerprinting

#### Manual clustering

For each song bout detected by field teams, we extracted corresponding recordings from the passive acoustic data using an automatic script in R v. 4.2.1^31^. These were then manually imported into Raven Pro v. 1.6 (Cornell Lab of Ornithology, USA), listened to, and visually inspected in the form of a spectrogram with a 1200-point Hann window (70% overlap) and a 2048-point Discrete Fourier Transform; brightness and contrast were initially set to 55 and 70, respectively, and adjusted if necessary. We found that individual male gibbons were readily-identifiable from recordings, even those from relatively poor-quality recordings (i.e., signal-to-noise ratios < 3 dB). Cao vit gibbon groups have a single adult male according to long-term monitoring data^14^; they alone contribute the male portion of the duets, although sub-adult males within a group may sing solo songs^14^. We therefore were able to assign each song bout to a particular group based on the male’s unique vocal fingerprint. We also took into consideration the location of each recording when determining the identity of a group, but this information was far less useful in most cases than the spectrogram.

#### Semi-supervised and unsupervised clustering

In addition to the manual vocal fingerprinting, we also explored more automated approaches to identifying how many males were present in the acoustic data. Specifically, we extracted measurements from each male phrase and then statistically clustered the data into groups. The number of resulting groups should equal the number of males present in the data. This more objective and repeatable approach was intended to complement the expert-driven manual clustering. Agreement between the two approaches might provide greater confidence in the overall population estimate.

To provide the data for clustering, we annotated the male multi-modulated phrases in each song bout recording with bounding boxes (Fig. 1). We only began annotating a given song bout once the male had started calling with the fully-developed form of his multi-modulated phrase (typically 8–10 min after the first call). Here, we did not annotate coda, staccato or boom phrases, although these may also encode information that is unique to each male. We also did not focus on female phrases, since previous work suggests that, for *Nomascus* gibbons, it is the males that are more easily individually identifiable^18,19^. Each bounding box was drawn to fully capture all parts of the phrase, including a small buffer of approximately 0.1 s either side of the call; if harmonics were visible, these were also included. During annotation, the number of ‘cao’ and ‘vit’ components of the phrase were also noted, defined as distinct frequency-modulated downsweeps and upsweeps, respectively (Fig. 1). We also included the first note of the multi-modulated phrase (sometime referred to as the ‘pre-modulated’ note^19^) in the count of the ‘cao’ component, even though in some males this was not heavily modulated in frequency. A simple count of the ‘cao’ and ‘vit’ components was chosen because of the ease with which it can be done, including in real-time in the field; indeed, field teams reported that they already used these characteristics to distinguish males. We also measured the 5th and 95th percentile frequencies in Raven Pro, as a robust index of the lowest and highest frequency of each phrase^21^, as well as the duration of each phrase.

Next, we extracted Mel-frequency cepstral coefficients (MFCCs), which allow for the rapid, standardised and automatic characterisation of acoustic data, including gibbon songs^32^. Following the methods in a previous study^32^, we extracted MFCCs in 12 Mel-frequency bins in the range 1–2 kHz across each of eight equal time windows for each phrase, using the R package ‘tuneR’ v. 1.4.1^33^. We discarded the first coefficient, since it is affected by signal power^32^, giving us 88 MFCCs. We also extracted an equal number of delta-cepstral coefficients, which help to characterise how a signal changes over time^32^. Combined with the features we measured in Raven Pro, we therefore had a total of 181 (= 5 + 88 + 88) measurements for each phrase.

We then used the measurements to cluster the male phrases into groups. For this, we used affinity propagation^34^ implemented in the R package ‘APCluster’ v. 1.4.10^35^, which has previously been used with success to cluster *Hylobates* gibbon songs^36^. We carried out the affinity propagation clustering in two ways: considering all labelled phrases and only considering ‘representative’ phrases for each male. Representative phrases were defined as phrases that had the modal number of ‘cao’ and ‘vit’ components for the given male. Given that this method required a first-pass identification of each phrase manually, we refer to this as a ‘semi-supervised’ clustering approach, as opposed to the unsupervised clustering we applied to the full dataset. The R packages ‘APCluster’ and ‘cluster’ v. 2.1.4 ^37^ were used to carry out ‘adaptive’ affinity propagation^36^ for the full dataset and the representative dataset, by tuning the value of the input preference parameter in order to maximise the average silhouette width across phrases. We visualised the clustering results with Uniform Manifold Approximation and Projection (UMAP), implemented in the R package ‘umap’ v. 0.2.9^38^.

### Estimation of total population size

Each of the clustering approaches—manual, semi-supervised and unsupervised—returned an estimate of the number of singing males that were detected during the population survey. In turn, this provided us with the number of family groups ($g$), after excluding any sub-adult males (which occasionally, but not always, sing at the same time as the adult male during song bouts). Since acoustic recorders were only operating in Vietnam during the population survey, we added to the estimated number of groups the three intensively-monitored groups that occur primarily in China (groups ‘GL’, ‘GM’ and ‘G1’)^15,39^.

An estimate of the total cao vit gibbon population size ($\widehat{N}$) was then obtained by summing the group sizes ${N}_{i}$ of groups that were successfully counted during the survey (${N}_{counted}= {\sum }_{i=1}^{{g}_{counted}}{N}_{i}$, where ${g}_{counted}$ is the number of successfully counted groups) and adding this to an estimate of the number of individuals present in the unobserved groups (${\widehat{N}}_{uncounted}$). We assumed that the average group size of successfully counted groups ($\widehat{s}$) could be used to estimate the number of unobserved individuals, i.e., ${\widehat{N}}_{uncounted}={g}_{uncounted}\widehat{s}$. The equation to calculate the population size was therefore: $\widehat{N} = {\sum }_{i=1}^{{g}_{counted}}{N}_{i} + {g}_{uncounted}\widehat{s}$. For the estimation of $\widehat{s}$, we only used counts from well-enumerated groups, i.e., counts from UAV-based observations or high-quality direct sightings where surveyors were confident that no gibbons were missed. Our estimate of the total population size excludes any solitary dispersing individuals (sometimes called ‘floating’ individuals^14^), which do not sing and are difficult to survey using any method. Long-term monitoring in China observed approximately one solitary male and female for each year of data^14^, suggesting low densities and-or low detection probabilities.

We incorporated uncertainty in $\widehat{s}$ using a Monte Carlo approach, wherein we drew samples (with replacement) from the observed group size counts to represent the unobserved groups and recalculated $N$ (n = 9999 simulations). A confidence interval on $\widehat{N}$ was obtained by taking the 95% quantiles over the resulting vector. Finally, we produced our ‘best’ estimate of the cao vit gibbon population, by incorporating group size information from long-term monitoring in Vietnam and China. In this case, we had group size information for all groups, so our estimate is considered exact (and therefore has no associated measure of uncertainty).

## Results

### Sampling effort

Between 26th October and 10th November 2021, we surveyed 29 listening posts across Vietnam and China, involving a total of 61 field personnel (42 in Vietnam and 19 in China) and 11 satellite campsites. Listening posts were surveyed for an average of 4.7 survey days (range: 1 to 9 days) and 28.9 h (range: 6 to 55 h). This generated 245 records of gibbons from field teams (Supplementary Figs. S1.1–S1.9). Following matching of data across survey teams, combined with vocal fingerprinting (see below), these records were deemed to have involved 49 song duets, 24 male solo songs and 28 direct observations. Most songs (55%) occurred in a 60 min period centred on sunrise (Fig S1.10).

The passive acoustic survey had a survey effort of 3480 days (1499 and 1981 in the first and second phases, respectively), generating more than 25,000 h of recordings. Each location was surveyed for an average of 63 days (range 4–84 days) and 471 h (range 29–632 h) over the two phases. Deployment and retrieval of devices during each phase involved a total of 11 personnel over 10 survey days.

### Measurement error and song density estimation

**Figure 3:**
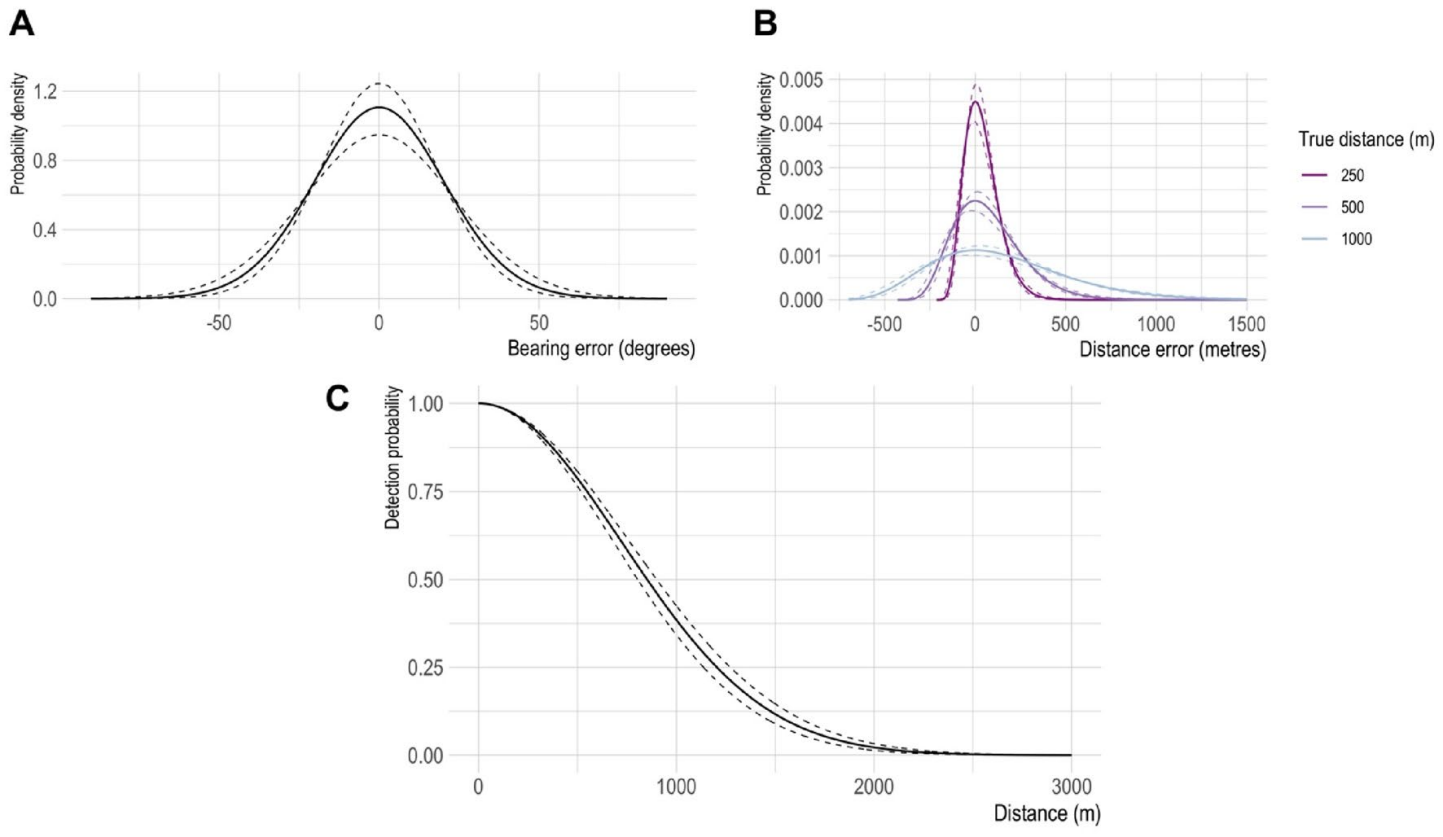
Estimated measurement error and detection range of surveyor teams when recording cao vit gibbon songs, as estimated using spatially explicit capture-recapture modelling. Measurement error is composed of (**A**) bearing error and (**B**) distance error, the latter of which is magnified when gibbons are far away. The detection range (**C**) was estimated using a half-normal detection function. Dashed lines indicate 95% confidence intervals.

SECR modelling revealed substantial human error associated with the distance and bearing measurements (Fig. 3a,b). For example, for a gibbon calling 500 m away, the 95% confidence interval (CI) on the distance measurement was estimated as 201–933 m. For a gibbon calling at 1000 m, the 95% CI was 402 to 1865 m. The 95% CI on bearing measurements was ± 41°. Surveyors had a greater than 90% chance of detecting singing gibbons at distances less than 330 m, but this had declined to 50% by 860 m (Fig. 3c).

Song density was better described by forest accessibility, as measured using distance from forest edge and elevation, than forest structure (ΔAIC_c_ =  − 38.3; Table 1); it was also better than a null model with no covariates (ΔAIC_c_ =  − 52.5). Forest that was further from the forest edge and at mid-elevation (i.e., on the mountain slopes, not on the highest peaks nor in the valleys) had substantially higher song density, with the confidence intervals on all parameters not overlapping zero (Table 1). Song density was also lower on rainy survey days, though this effect was not statistically clear, with the confidence interval on the parameter overlapping zero (Table 1); indeed, gibbons were recorded singing on days when it rained. Gibbons were predicted to sing mostly within a highly restricted area of the forest, greater than 1 km from the forest edge and between 750 and 790 m elevation (Supplementary Figs. S1.11–S1.12).

**Table 1:** Spatially explicit capture-recapture parameter estimates for models relating song density (songs per hour of survey per km^2^) to covariates. The detection model parameters for the forest accessibility and forest structure models were similar and we here only report those from the accessibility model. Covariates for which the confidence intervals do not overlap zero are considered to be statistically clear results.

Parameter	Parameter estimate	Standard error	95% confidence interval
**Forest accessibility model for song density (AIC**_**c**_ *= 4332.09)*			
Intercept	− 13.11	2.47	− 17.94 to − 8.27
Distance from forest edge	0.56	0.14	0.29–0.82
Elevation	32.91	10.55	12.23–53.59
Elevation^2^	− 36.03	11.01	− 57.61 to − 14.44
Weather (rain)	− 0.28	0.25	-0.77–0.20
**Forest structure model for song density (AIC**_**c**_ **= 4370.46)**			
Intercept	− 8.24	0.43	− 9.09 to − 7.39
Tree canopy cover	1.52	0.47	0.59–2.45
Tree canopy height	0.45	0.37	− 0.27–1.17
Weather (rain)	− 0.26	0.25	− 0.74–0.23
**Detection model**			
Detection function intercept (g_0_)	1 (fixed)	–	–
Detection function scale (σ)	734.81	20.93	693.78–775.83
Distance measurement error (ɑ)	7.00	0.78	5.48–8.53
Bearing measurement error (κ)	7.96	1.04	5.92–10.00

### Manual and automatic clustering of the song bouts

By matching the song bout data from field teams with the acoustic data, we were able to extract recordings of 55 song bouts, over which we annotated 940 multi-modulated phrases. We deduced from the field data that two of the song bouts were of a group (‘GL’) located far on the Chinese side of the border, more than 1 km from our devices; we excluded these from further analysis due to the low quality of the recordings.

**Figure 4:**
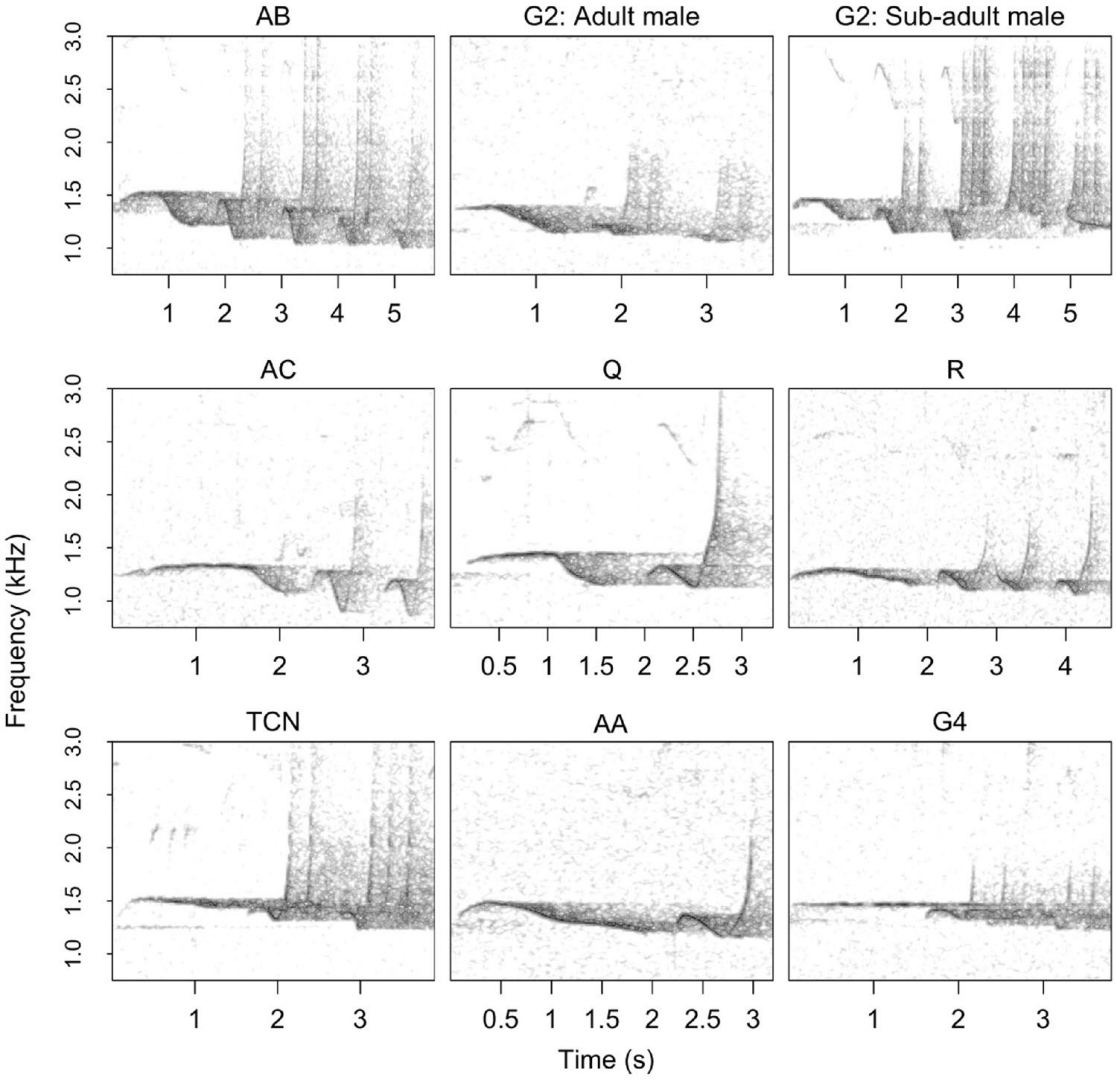
Example multi-modulated phrases for the nine manually-identified male cao vit gibbons. Spectrograms were created with the ‘warbleR’ package v. 1.1.28 in R^47^, using a 1200-point Hann window with 70% overlap.

**Figure 5:**
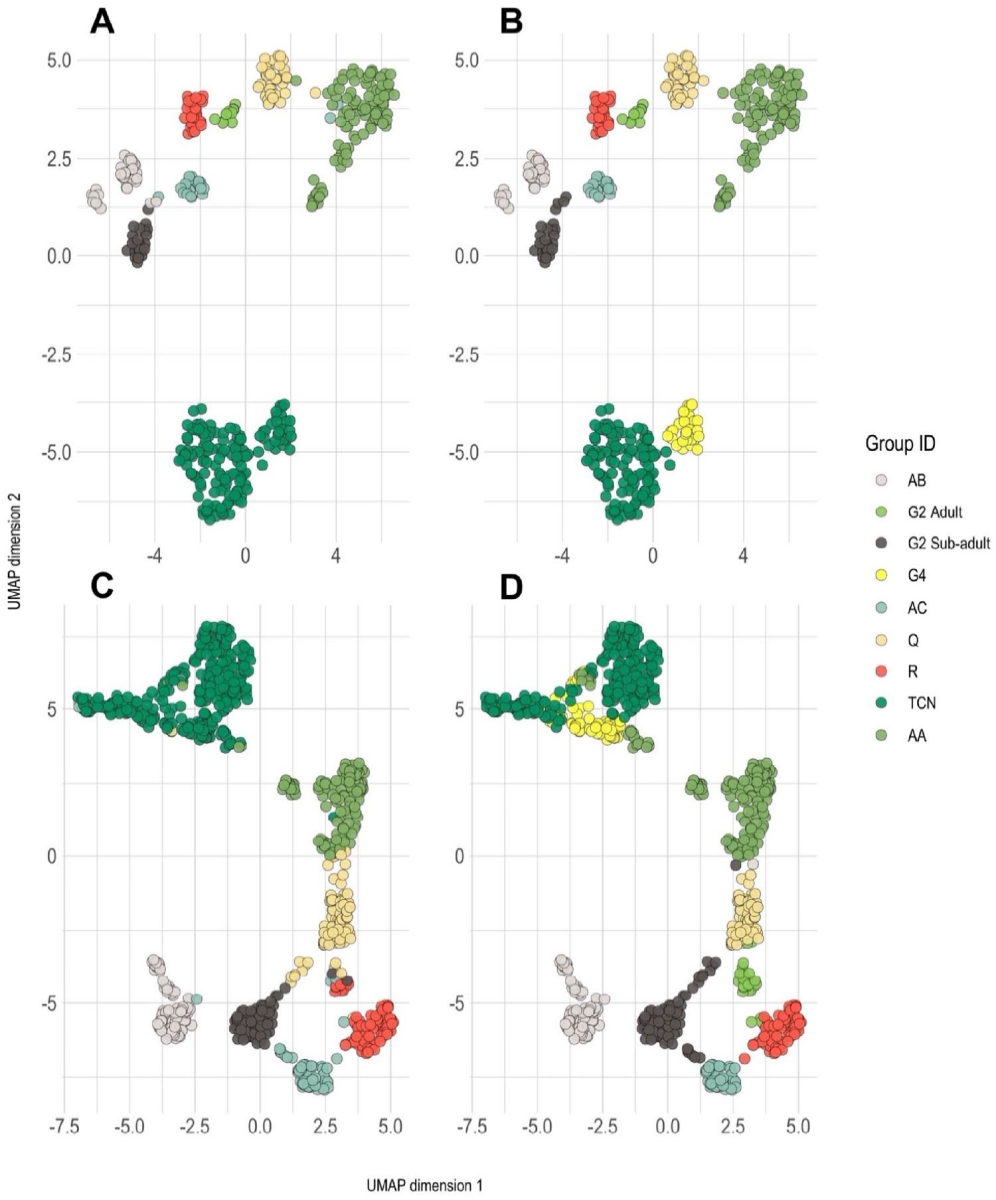
Clustering of the cao vit gibbon male calls based on affinity propagation (**A**, **C**) and compared to a manual approach (**B**, **D**). Each male phrase is plotted in two-dimensional space by applying Uniform Manifold Approximation and Projection (UMAP) dimension-reduction to 181 different measurements. Top row: Semi-supervised affinity propagation applied to the ‘representative’ phrases (**A**) as compared to the manual clustering (**B**). Bottom row: Unsupervised affinity propagation applied to the full dataset (**C**) as compared to a manual approach (**D**).

For the remaining 53 song bouts, manual clustering revealed nine different singing males across eight family groups (Fig. 4). One of the groups, G2, was represented by two males: an adult and putative sub-adult. The sub-adult often sang alone and was only ever heard singing with the G2 females alongside the adult male. It is also possible that this individual was an unmated adult male challenging the established G2 male. Monitoring of G2 for a longer period than we could achieve during our survey would help to clarify the relationship between this sub-adult and group G2. In any case, the number of singing family groups remains eight. Five other male solo songs were recorded but only involved simple, undeveloped phrases that could not be identified. These songs were excluded from further analysis but, based on the locations of these solo songs, we estimate that they involved three different males; solitary dispersing males are not thought to sing^14^, so it is likely that these males were part of known family groups. Each of the nine manually-identified clusters were discernible in a UMAP plot of the acoustic features (Fig. 5b,d).

Affinity propagation clustering using the semi-supervised approach returned eight clusters across seven family groups (Fig. 5a), whilst the unsupervised approach returned seven clusters across seven family groups (Fig. 5c). Compared to the manual identification, the semi-supervised and unsupervised approaches lumped together groups TCN and G4. The unsupervised approach also did not resolve the G2 adult male as a distinct cluster and the G2 sub-adult male instead represented group G2 in this case.

### Population size estimation

**Figure 6:**
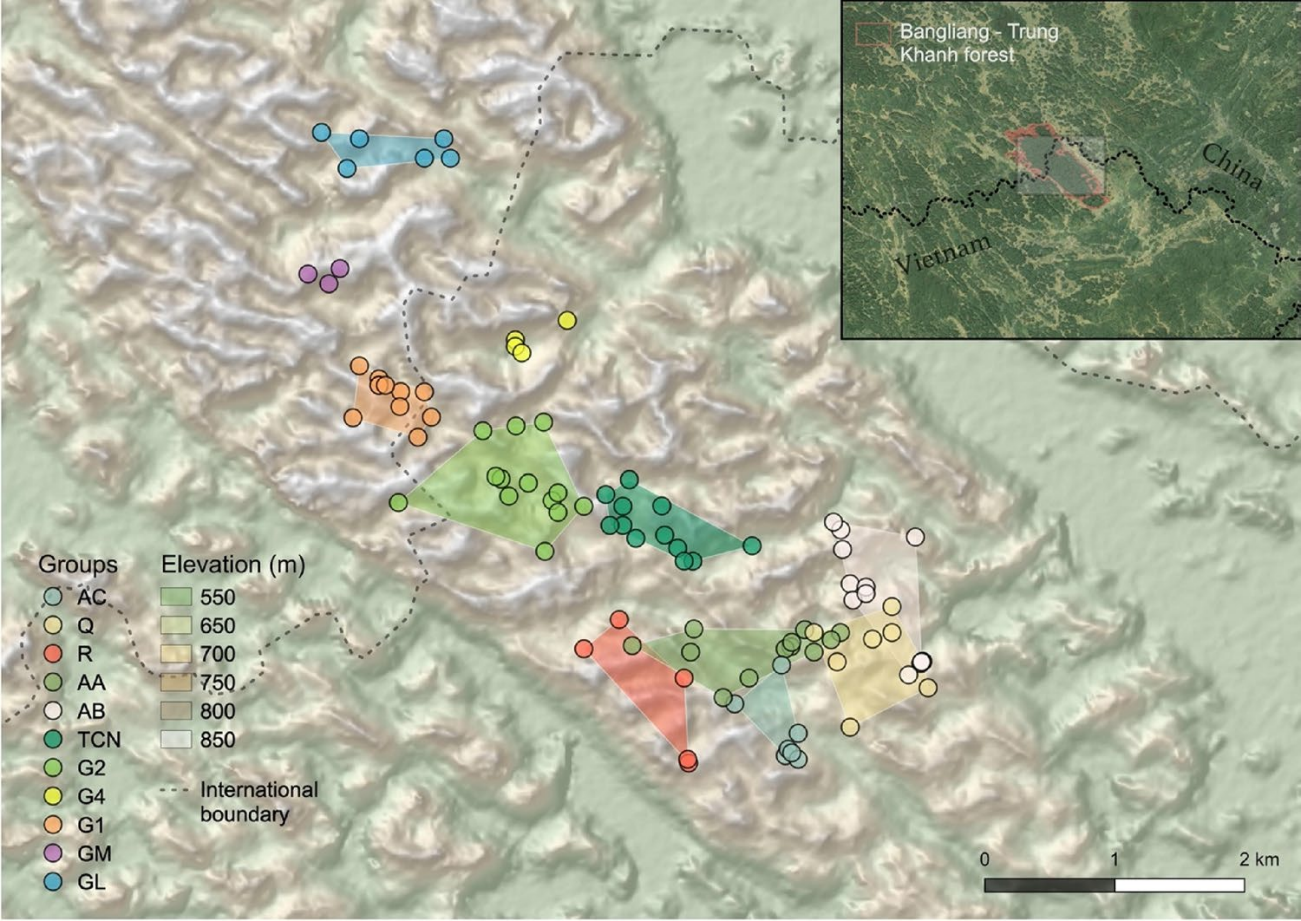
Gibbon detection locations (songs and sightings) and minimum convex polygons for groups with sufficient data (n ≥ 5). Song locations were estimated using spatially explicit capture-recapture modelling, based on distance and bearing measurements from survey teams, whilst direct observations were mapped in the field based on topographical landmarks. The inset map shows the broader landscape surrounding the Bangliang—Trung Khanh forest block (Google Earth basemap).

On the basis of the manual clustering and the group size counts made during the survey (direct observations and UAV videos), we estimate that there were 11 cao vit gibbon family groups, comprised of 76 individuals (95% CI: 74–78; Table 2). By incorporating long-term monitoring data to fill in data gaps about group sizes (for groups Q, G4 and GL), we estimate that there were in fact 74 individuals in family groups. The semi-supervised and unsupervised approaches both produced an estimate of 10 family groups, comprised of 69 individuals in both cases (95% CI: 67–70). Three of the groups primarily resided in China and eight in Vietnam, with considerable overlap observed in the home ranges of the southern-most groups in Vietnam (Fig. 6).

**Table 2:** Cao vit gibbon groups identified from vocal fingerprinting of acoustic data collected from September to October 2021. The composition of each group was derived from survey data (direct sightings or UAV videos) or, for groups Q, G4 and GL, from long-term monitoring data. The total population size does not include any solitary dispersing individuals, which are difficult to survey. Kernel home ranges are indicative only.

Group name	Core range (valley names)	Composition					Total individuals	Number of duets (*p*_daily_)		Minimum convex polygon (ha)	95% kernel home range (ha)
		Adult females	Adult males	Sub-adults	Juveniles	Infants		Block 1 survey (4 days)	Block 2 survey (5 days)		
*AC*	Đắc, Cô 1	2	1	1	2	1	7	1 (0.2)	–	17.7	128.6
*Q*	Tâm Đeng, Tậu Lô	2	1	0	1	1	5	3 (0.8)	–	49.2	153.1
*R*	Cô 2, Thềnh	3	1	0	1	1	6	2 (0.5)	–	36.6	240.6
*AA*	Toong On, Đắc Roong	2	1	1	2	1	7	4 (0.8)	5 (1.0)	46.0	123.2
*AB*	Táp Toan nhỏ, Hoai	2	1	1	2	1	7	6 (1.0)	4 (0.6)	47.3	125.4
*TCN*	Nguốc Mần, Đẩy	2	1	1	2	1	7	4 (0.8)	3 (0.6)	33.8	85.6
*G2*	Boỏng Bíp, Kỳ Già	2	1	1	3	0	7	–	2 (0.4)	81.5	181.7
*G4*	Gu, You	2	1	1	2	1	7	–	2 (0.4)	–	–
*G1*	Hao, Gong	2	1	2	3	0	8	–	8 (0.8)	21.1	54.1
*GM*	Kou	2	1	0	2	1	6	–	1 (0.2)	–	–
*GL*	Paisa, Long	2	1	0	2	2	7	–	4 (0.8)	18.8	147.5
Total individuals (including long-term data)		23	11	8	22	10	74				
Total individuals (survey data only)							76 (95% CI: 74–78)				
Total individuals (semi- and un-supervised clustering)							69 (95% CI: 67–70)

Group compositions observed during the survey were consistent with those from long-term monitoring in China, with most groups consisting of one adult male, two adult females and dependent offspring (Table 2). The exception was group R, which apparently had three adult females (though, consistent with our understanding of the species, this is unlikely to be stable in the long-term^14^).

## Discussion

We carried out the most robust survey of the last remaining cao vit gibbon population done to date, incorporating vocal fingerprinting and UAV-based group counts, finding that the population is substantially smaller than previously thought. Instead of approximately 120 individuals, the population is 38% smaller, at 74 individuals (plus an unknown number of solitary dispersing individuals). Semi-supervised and unsupervised approaches to clustering the acoustic data yielded a slightly smaller population size estimate of 69 individuals, with no evidence that the manual identifications had missed any groups.

Previous surveys, we believe, have unwittingly over-estimated the population size by occasionally double-counting groups when they sang in new locations (either on the same day or subsequent days). The double-counting problem has likely been exacerbated by the substantial measurement error in estimated distances and bearings, causing inaccurate localisation of singing gibbons. We saw evidence of this double-counting during our survey, and instead relied on the acoustic data to decide when to split or combine records. We have no evidence that the discrepancy in estimates between this latest survey and previous surveys is due to a population decline, with no hunting reported over the last 20 years and the habitat undergoing recovery since at least 2007^3^. Indeed, we believe the population has likely increased over this time, with two new groups having formed in China (in 2015 and 2017) in previously unoccupied habitat^15,39^.

The cao vit gibbon is evidently in much more immediate danger from small population size effects—including loss of genetic diversity, inbreeding, and vulnerability to unforeseen catastrophes—than previously thought^3^. There are at least three implications of this new understanding of the population size. First, there is now an even greater urgency to the ongoing habitat restoration work, likely the most feasible way to increase the population size over the near-to-medium term. Habitat restoration in limestone forest has proven challenging to date, owing to the unique ecology of succession in this habitat and the difficult access to the site^40^. Fauna & Flora are currently trialling new methods, such as soil transplants in rocky areas and cluster planting in valley bottoms, but additional expertise and resources are needed. In China, too, habitat restoration efforts are underway^3^ and the potential gains for the population are even larger than in Vietnam: as much as 84% of the forest block remains unoccupied by gibbons on the Chinese side of the border, compared to 73% in Vietnam (based on a minimum convex polygon around the detection locations and long-term data^9^). This difference is largely due to the highly degraded state of the habitat in the extreme northwest of the forest block in China^12^.

The second implication of the new, substantially smaller population estimate is that there is an even stronger rationale to continue the monitoring of focal groups into the long-term, as an early-warning system to detect inbreeding depression (as might be indicated, for example, by changes in infant mortality rates or female breeding rates). Long-term monitoring also appears to provide de facto protection for monitored groups, due to the regular presence of monitoring teams in the field.

The third implication of discovering the very small size of the gibbon population is that it calls into question current plans to reintroduce the species to an additional site^3^, since any removal of individuals from the population may endanger its persistence more than previously thought. Careful study of the vital rates (breeding and mortality) of the existing population, as well as updated scenario modelling using population viability analysis (PVA)^3,12^, will be needed in order to assess the feasibility and risks associated with this. As a crucial input to the PVA modelling, the genetic health of the population must urgently be assessed, as has been done for example for the Hainan gibbon (*N. hainanus*)^41^. The genetic assessment would also help to define the time-line over which conservation actions must occur.

We consider it unlikely that we have missed any groups during our survey, due to the high sampling effort in the field, specifically the high density and coverage of listening points used. Unsurveyed areas within the Bangliang—Trung Khanh forest block, for example to the north and south of known gibbon groups (Fig. 6), are unlikely to harbour any further groups, with patrols and local communities regularly visiting these areas but never reporting gibbon songs or sightings. It is possible, though, that these areas harbour dispersing gibbons, which are typically silent and therefore highly cryptic. Our new estimate is also more plausible than previous estimates, since it resolves the discrepancy in the population data from China and Vietnam. The estimated density in Vietnam of 1.4 groups per km^2^ (six Vietnam-only groups occupying 4.4 km^2^; calculated from a minimum convex polygon around the detection locations) is now more in line with the density from long-term monitoring in China of 1.0 groups per km^2^.

The main caveat to our vocal fingerprinting approach is that it may fail to distinguish individuals if songs show a high degree of similarity. Indeed, the semi-supervised and unsupervised approaches both lumped together some males that the manual process determined were separate. We consider it unlikely, however, that the manual approach missed any males. As well as the multi-modulated phrases, the manual approach also used information from the staccato phrases (which appeared to differ in shape and peak frequency between males) and geographic location and timing of calls. Previous studies of *Nomascus*^18,19^ and *Hylobates*^21^ have also not uncovered any evidence of cryptic individuals. Nonetheless, it may be beneficial to apply more sensitive approaches to classifying the acoustic data than affinity propagation, in particular convolutional neural networks^42^. These algorithms rely on manually-generated training data, but songs which the algorithm finds difficult to classify (i.e., with low class probabilities) might indicate the presence of cryptic individuals. These songs could be flagged and investigated further.

SECR has been applied to relatively few gibbon species thus far^25,43^, perhaps in part due to the statistical complexity of the approach relative to traditional triangulation methods. However, the SECR approach provides a rigorous way of reconciling bearing and distance measurements from different surveyor teams and estimating the location of singing gibbons. It also quantifies the magnitude of the measurement errors which, for the cao vit gibbon, we found were substantial (Fig. 3), most likely due to the complex way in which sound travels in the karst mountain landscape. SECR modelling can also be used to test hypotheses, such as the drivers determining singing locations. For the cao vit gibbon, we found that highly specific locations within the Bangliang—Trung Khanh forest are favoured for singing: upper mountain slopes far from the forest edge (Supplementary Fig. S1.12). The elevation effect is similar to findings from intensive, long-term monitoring of two cao vit gibbon groups in China, which found that gibbons preferred higher elevations for singing^9^. In principle, SECR could also be used with capture-recapture data from passive acoustic monitoring arrays, outputting the estimated locations of singing gibbons. For accurate localisation, however, supplementary information is likely needed, such as bearings (e.g. from a recorder capable of carrying out beamforming), signal strength and-or time-of-arrival (e.g. from devices that are time-synchronised using GPS)^44–46^.

Our approach in the 2021 survey, which combined traditional methods with emerging, technology-based methods, paves the way for a new era in monitoring of the cao vit gibbon, and indeed other gibbon species. Gibbon surveys employing vocal fingerprinting should be more accurate and comparable across time. Moreover, since male *Nomascus* songs are stable over long time-frames^18,19^, it is likely possible to match individual males over consecutive surveys and detect male replacement events at the population scale^19^. In the long-term, we might even envisage a solar-powered, time-synchronised acoustic array, combined with edge processing and cellular connectivity, that could autonomously map the songs of different males and send the information remotely. With this, we could move away from snapshot population surveys and instead monitor the population continuously across space and time. This would provide conservation managers with an unprecedented level of detail about gibbon populations and allow for more effective and timely decision-making.

### Supplementary Information

Supplementary Information.

## Data Availability

The acoustic measurements (which were input to the clustering analyses) are publicly available on the Zenodo repository at the following URL: https://zenodo.org/doi/10.5281/zenodo.10138012. We have not made the detection data from the survey (beyond those already presented in the tables and figures) publicly available, due to the highly threatened status of the study species. Data requests can be sent to the corresponding author and access will be granted in all reasonable cases.
