# Supplementary material for: Vocal fingerprinting reveals a substantially smaller global population of the Critically Endangered cao vit gibbon (Nomascus nasutus) than previously thought
Source: Sci Rep. 2024 Jan 3;14:416. doi: 10.1038/s41598-023-50838-2 (PMC10764777; doi:10.1038/s41598-023-50838-2)

Supplementary Information for: Wearn *et al.* 2023. Vocal fingerprinting reveals a substantially smaller global population of the Critically Endangered cao vit gibbon (*Nomascus nasutus*) than previously thought. *Scientific Reports*. https://doi.org/10.1038/s41598-023-50838-2.

Contents:

Supplementary Figures S1.1-S1.12.

Figures S1.1-S1.9. Song bout records from field teams during the 2021 cao vit gibbon population survey. Song bouts were matched on the basis of bearing, distance and time of day, as well as vocal fingerprinting from acoustic recordings. Arrows depict the bearings and distances reported by survey teams stationed at survey posts (black points). The survey was done in two phases: Phase 1 in the southern half of the study area from 27th to 31st October and Phase 2 in the northern half of the study area (including China) from 6th to 10th November. Rain on the 29th October meant that no gibbons were detected on this day and this survey day is omitted here. The basemap is a greyscale hillshade of elevation with contours (20 m spacing) overlaid.

Figure S1.1. 27th October 2021.

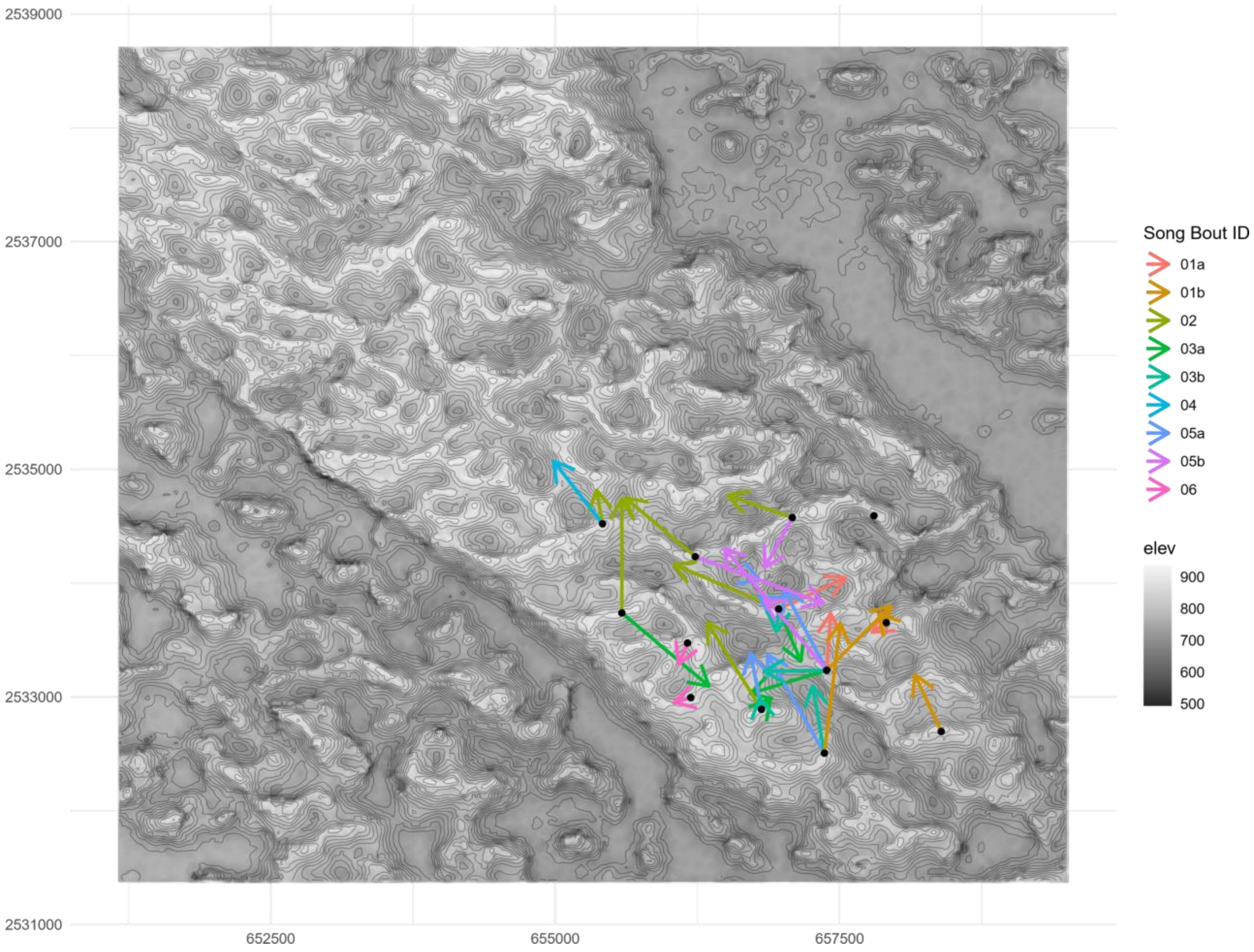

Figure S1.2. 28th October 2021.

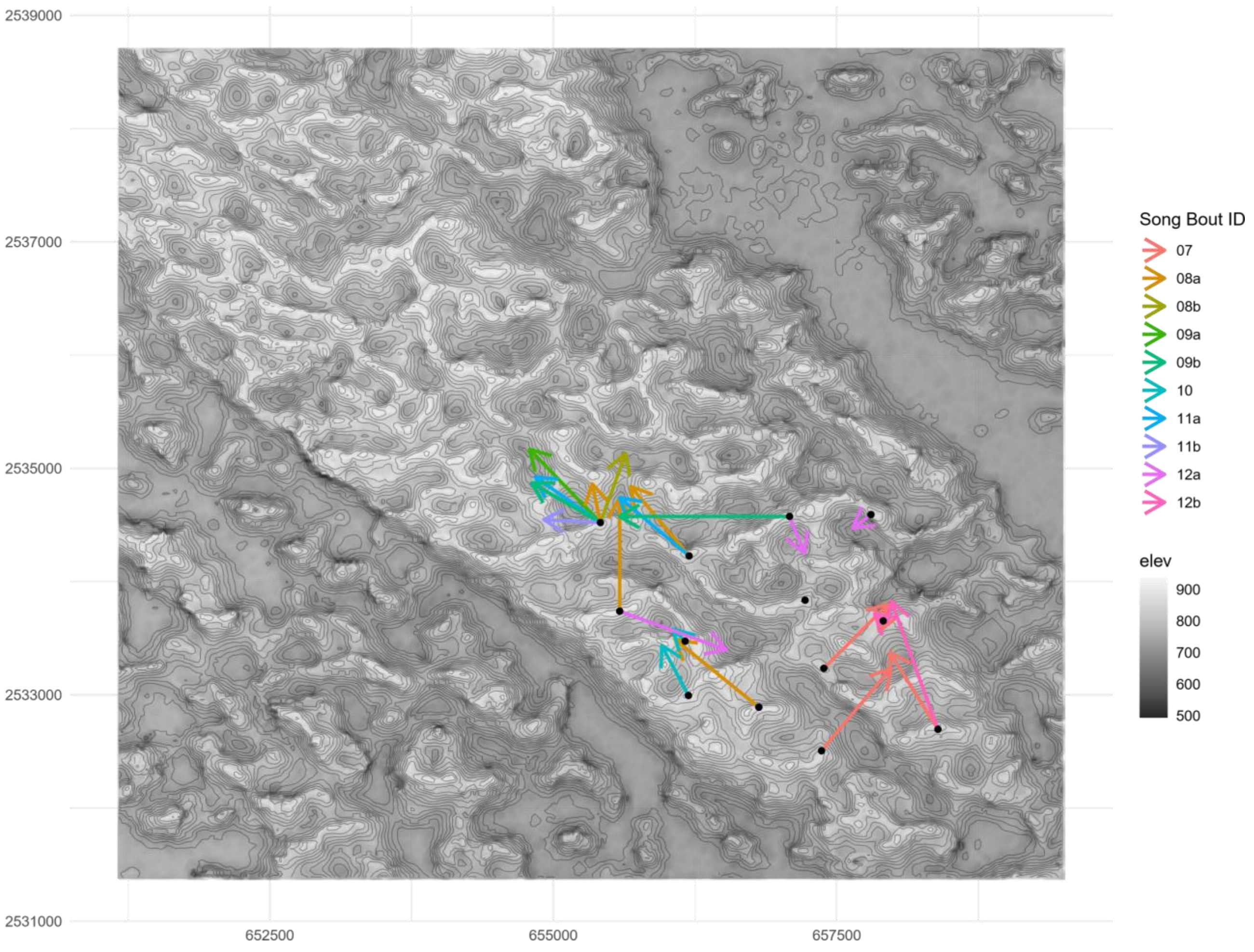

Figure S1.3. 30th October 2021.

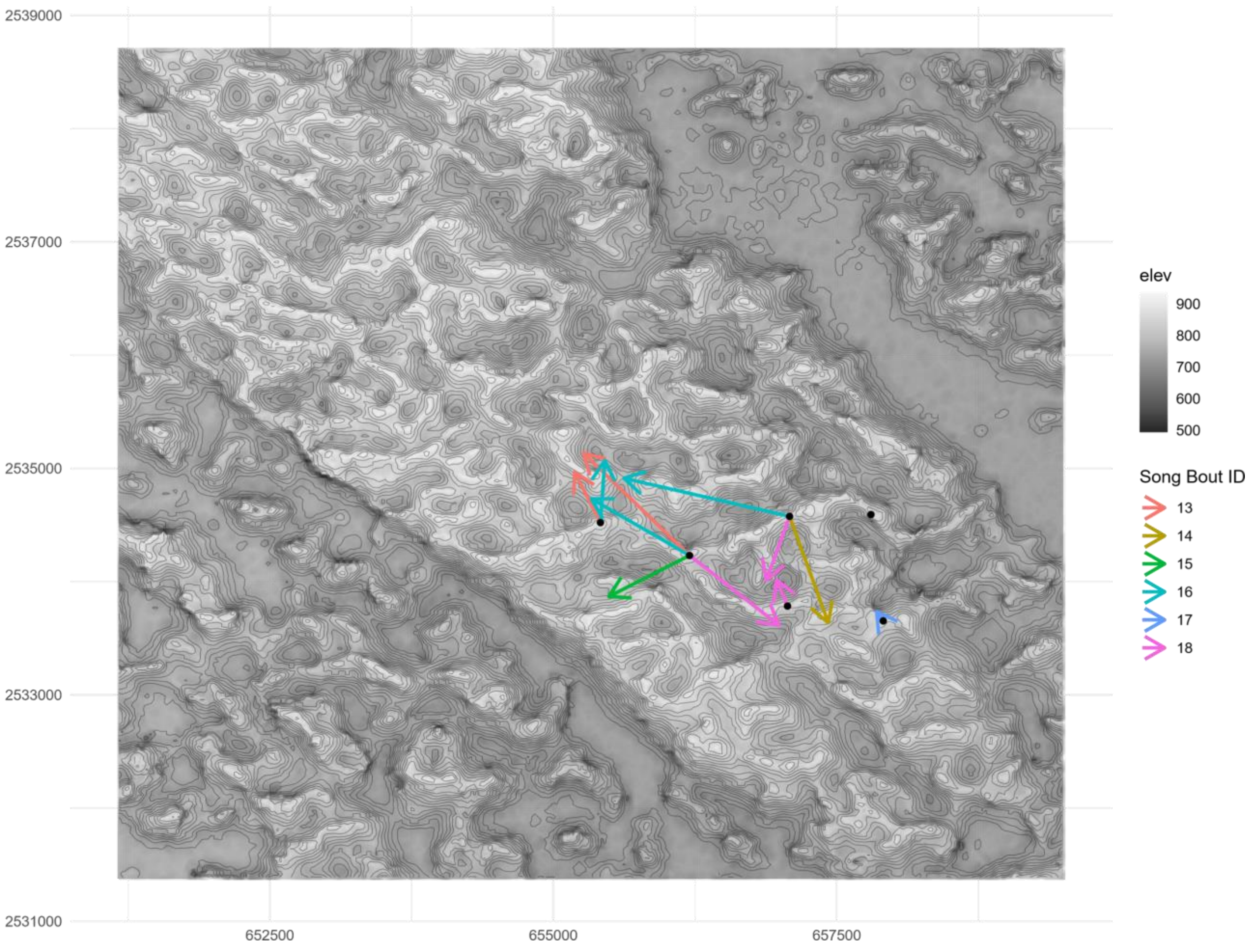

Figure S1.4. 31st October 2021.

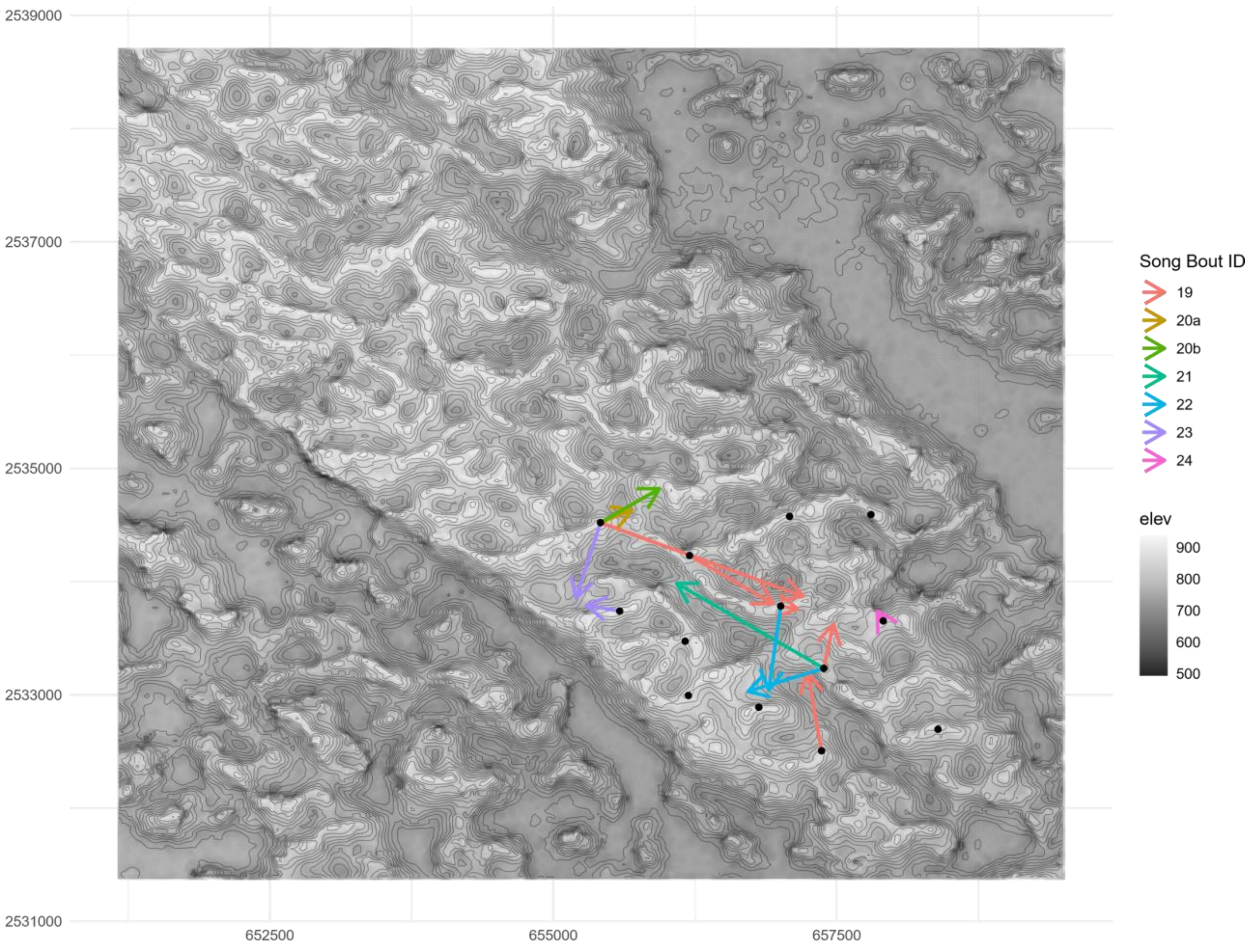

Figure S1.5. 6th November 2021.

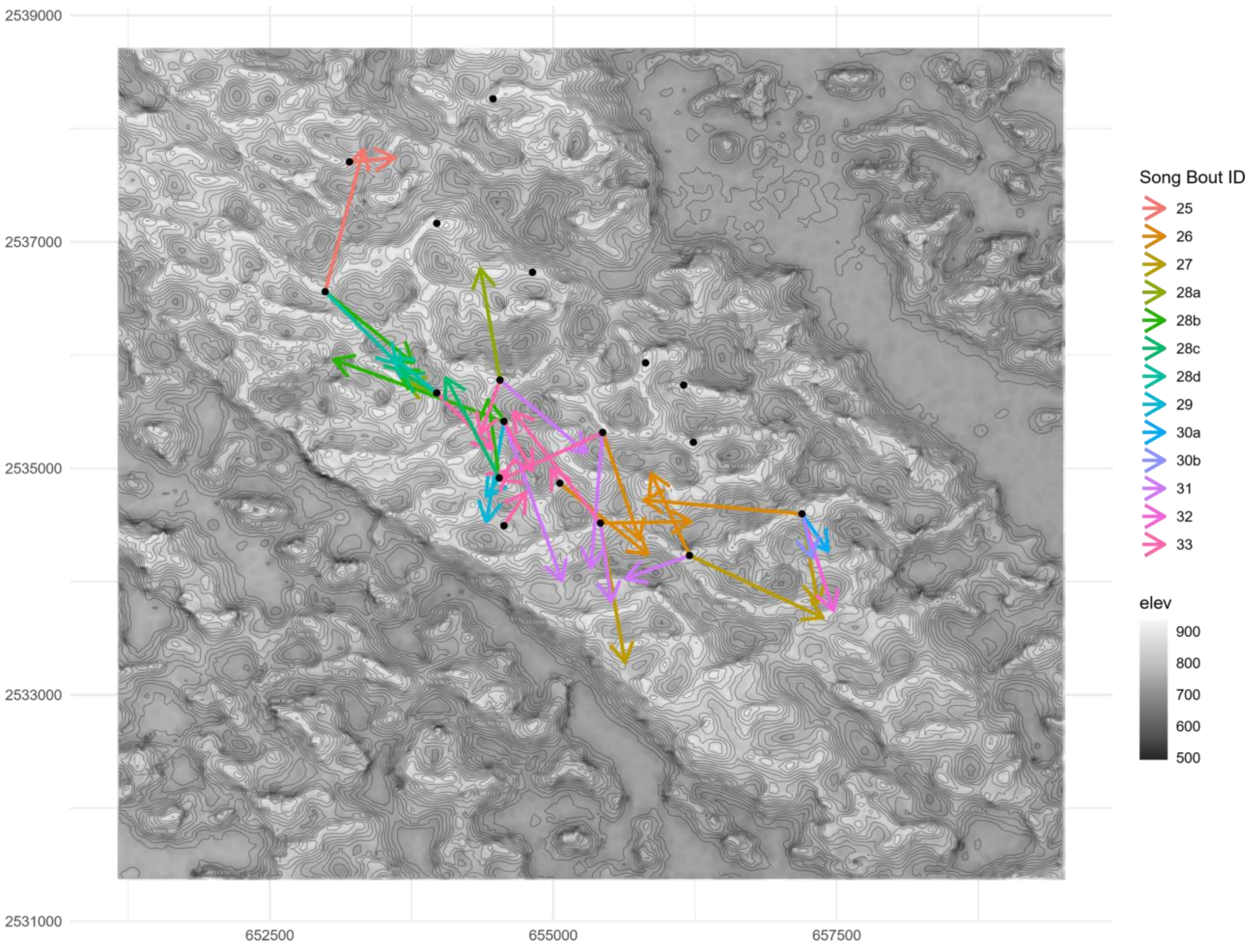

Figure S1.6. 7th November 2021.

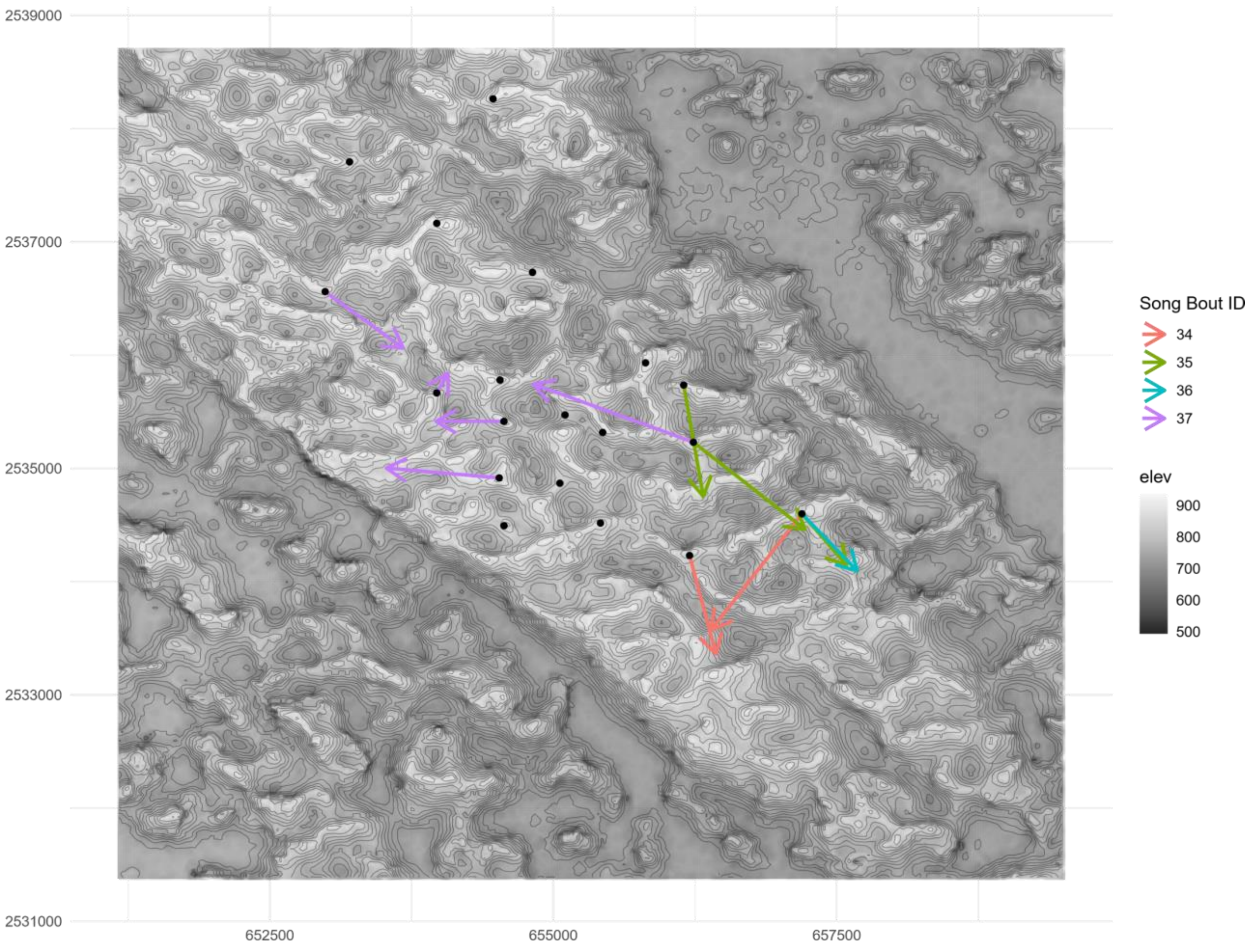

Figure S1.7. 8th November 2021.

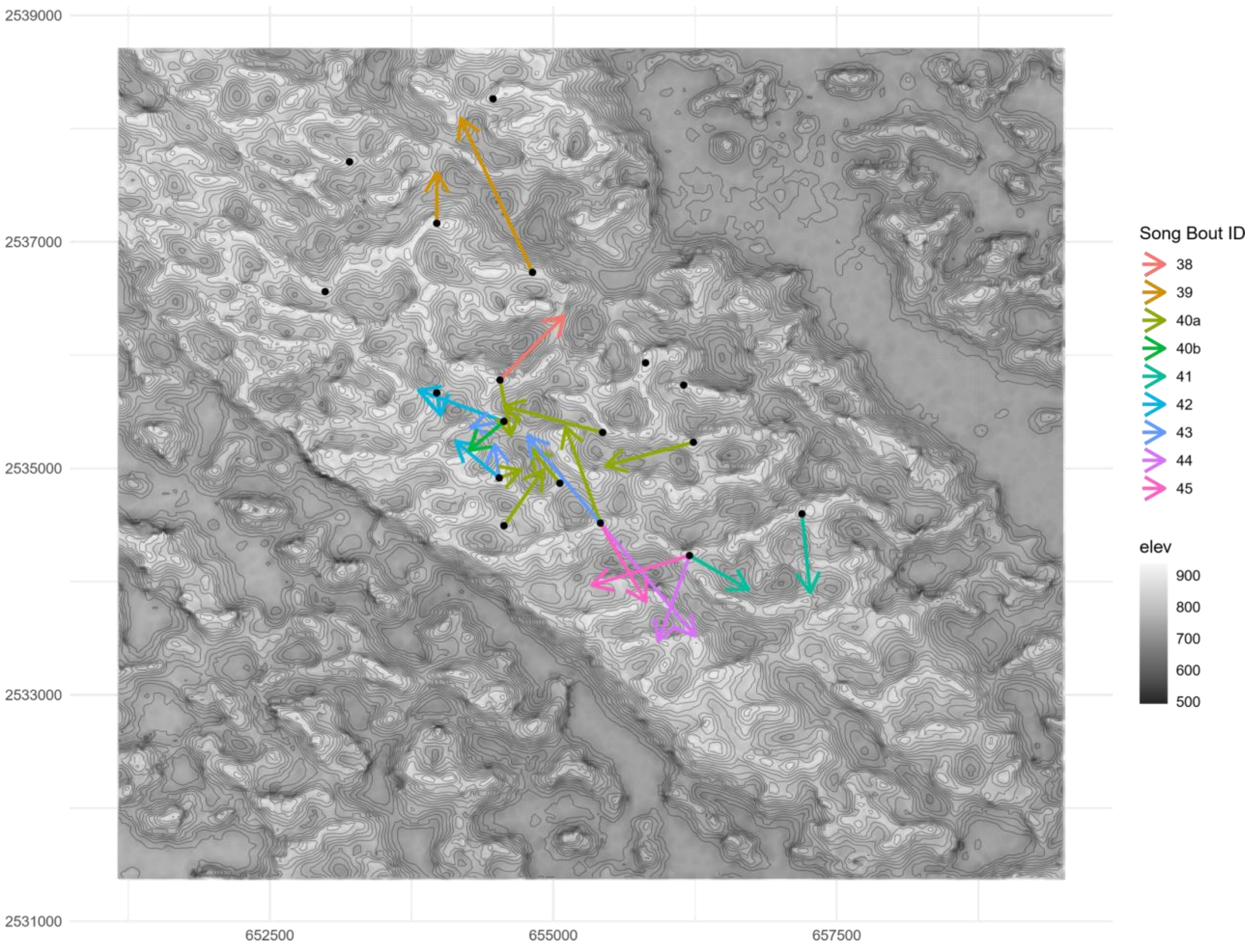

Figure S1.8. 9th November 2021.

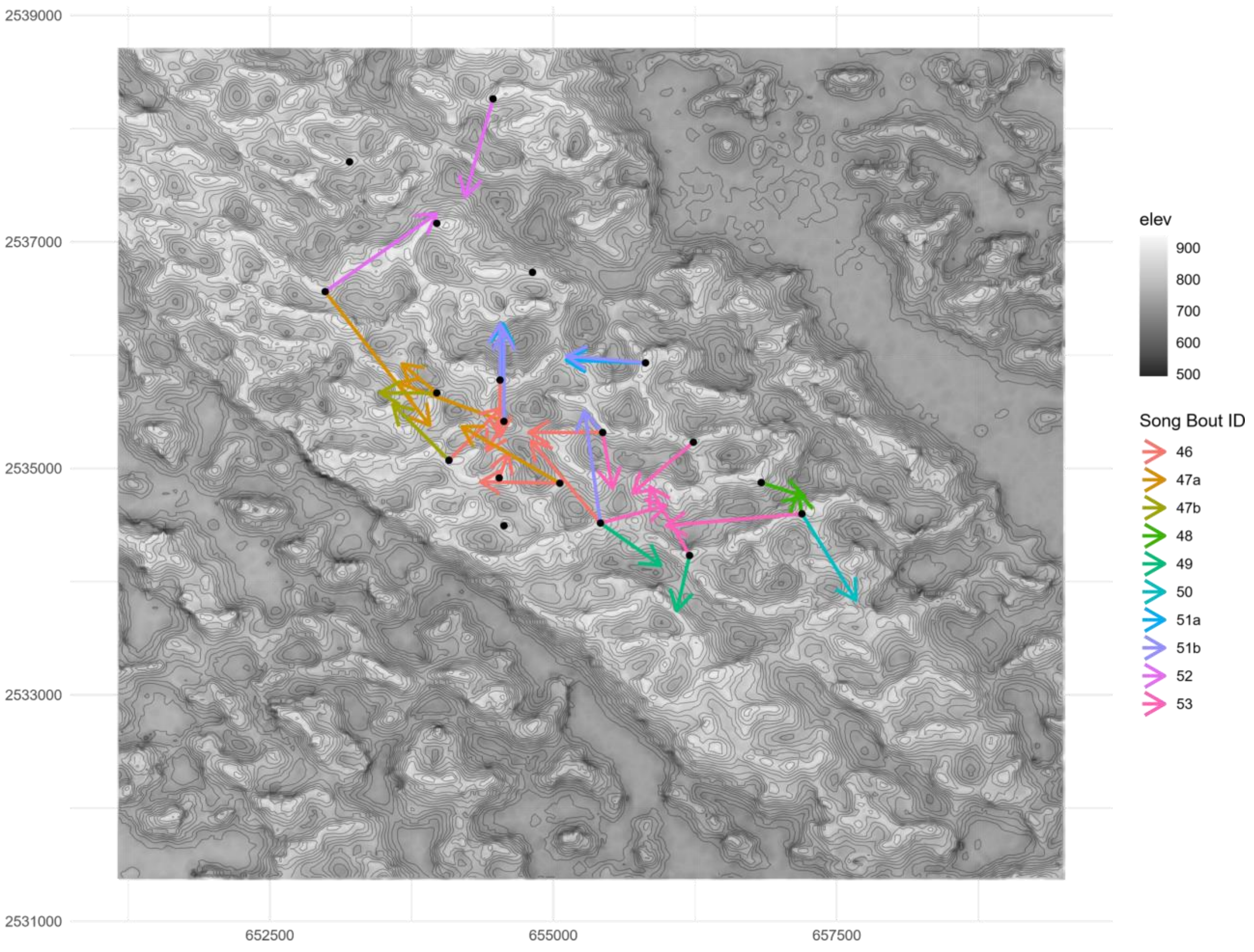

Figure S1.9. 10th November 2021.

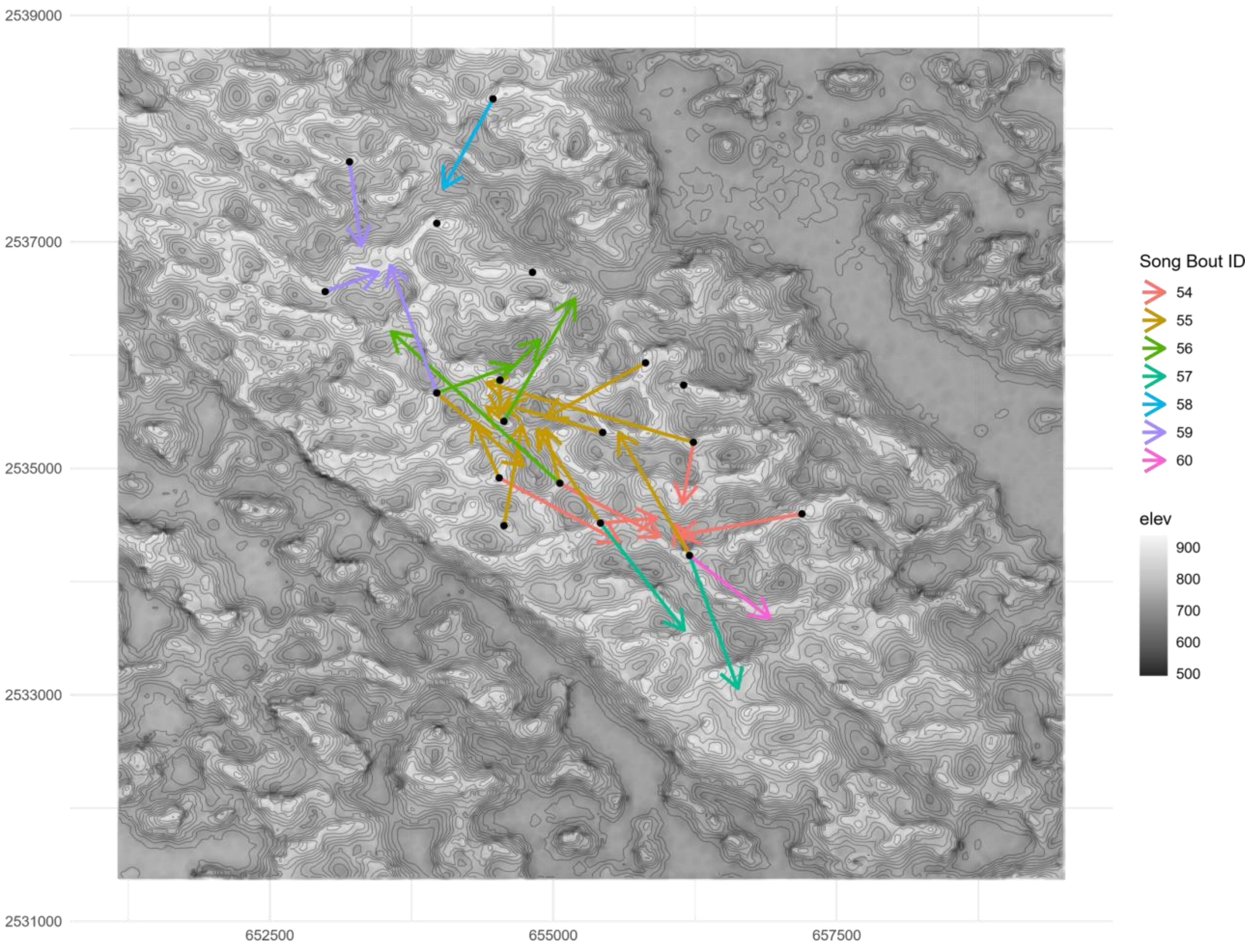

Figure S1.10. Distribution of song bout start times recorded by field teams during the 2021 cao vit gibbon population survey. The peak of singing was at 06:00 am, which corresponds exactly to sunrise time (05:56 – 06:04 am during the survey).

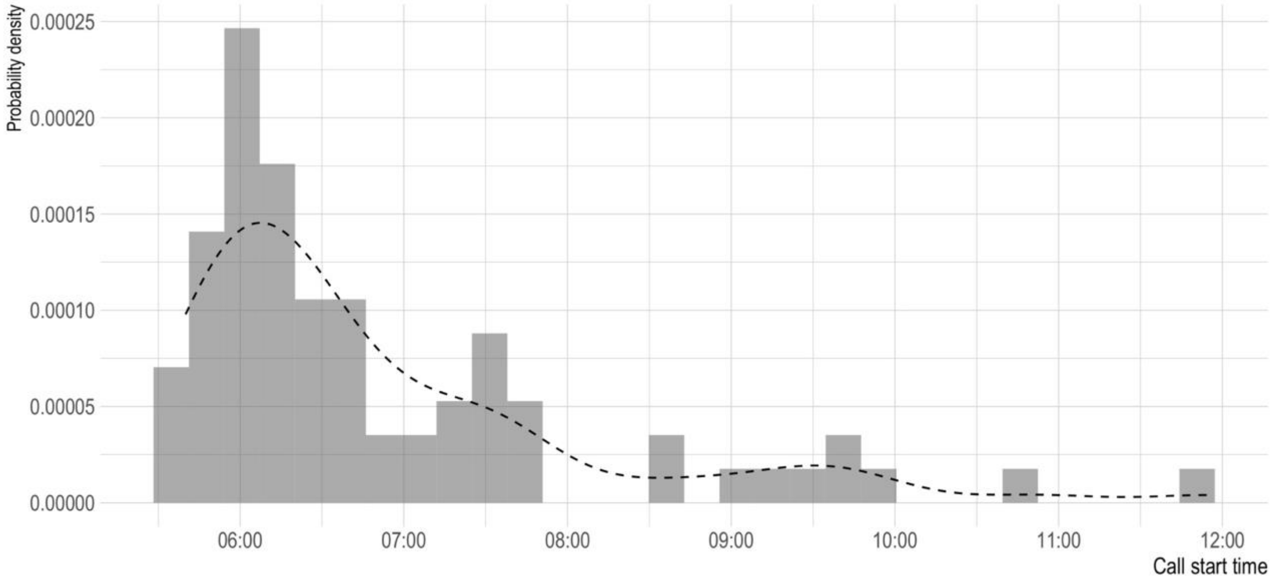

Figure S1.11. Important drivers of spatial variation in song density during the 2021 cao vit gibbon population survey, as revealed by spatially explicit capture-recapture modelling. (A) Predicted song density as a function of elevation, with distance from edge fixed at 1,250 m. (B) Predicted song density as a function of distance from forest edge, with elevation fixed at 776 m (the average elevation for the area occupied by gibbons).

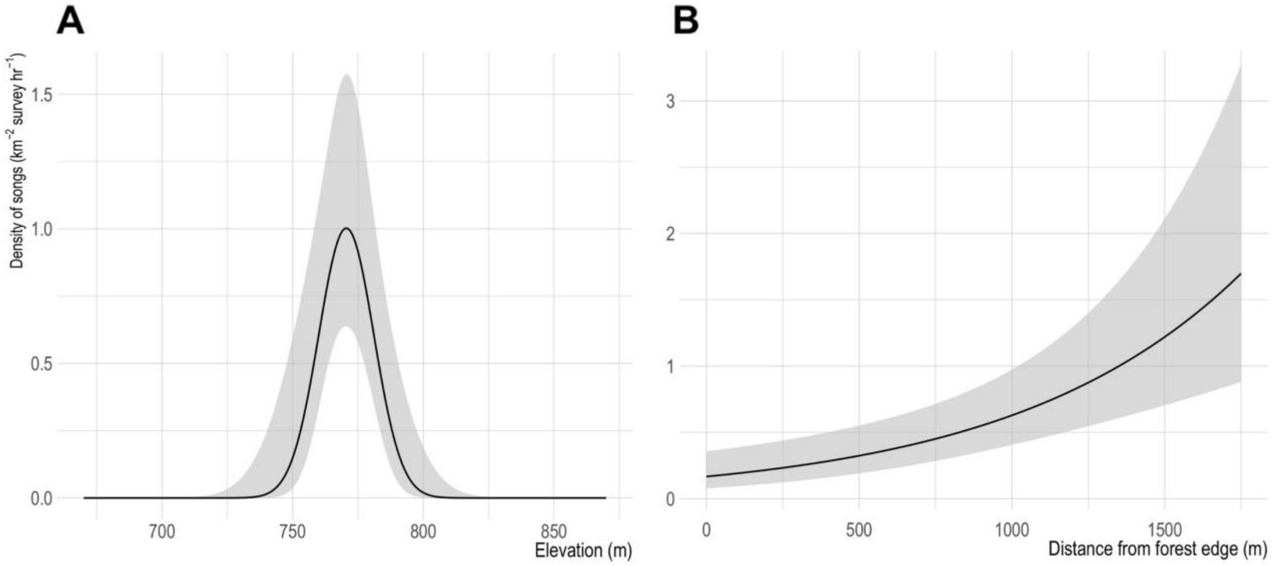

Figure S1.12. Predicted song density based on spatially explicit capture-recapture modelling of field data. Predictions were generated using forest accessibility variables (elevation and distance from forest edge). Areas with a song density less than 0.2 km^{-2} survey hr^{-1} are not shown. Song bout locations are those estimated from the model. Basemap is a greyscale hillshade of elevation. Inset map shows the location of the study area in northern Vietnam – southern China.

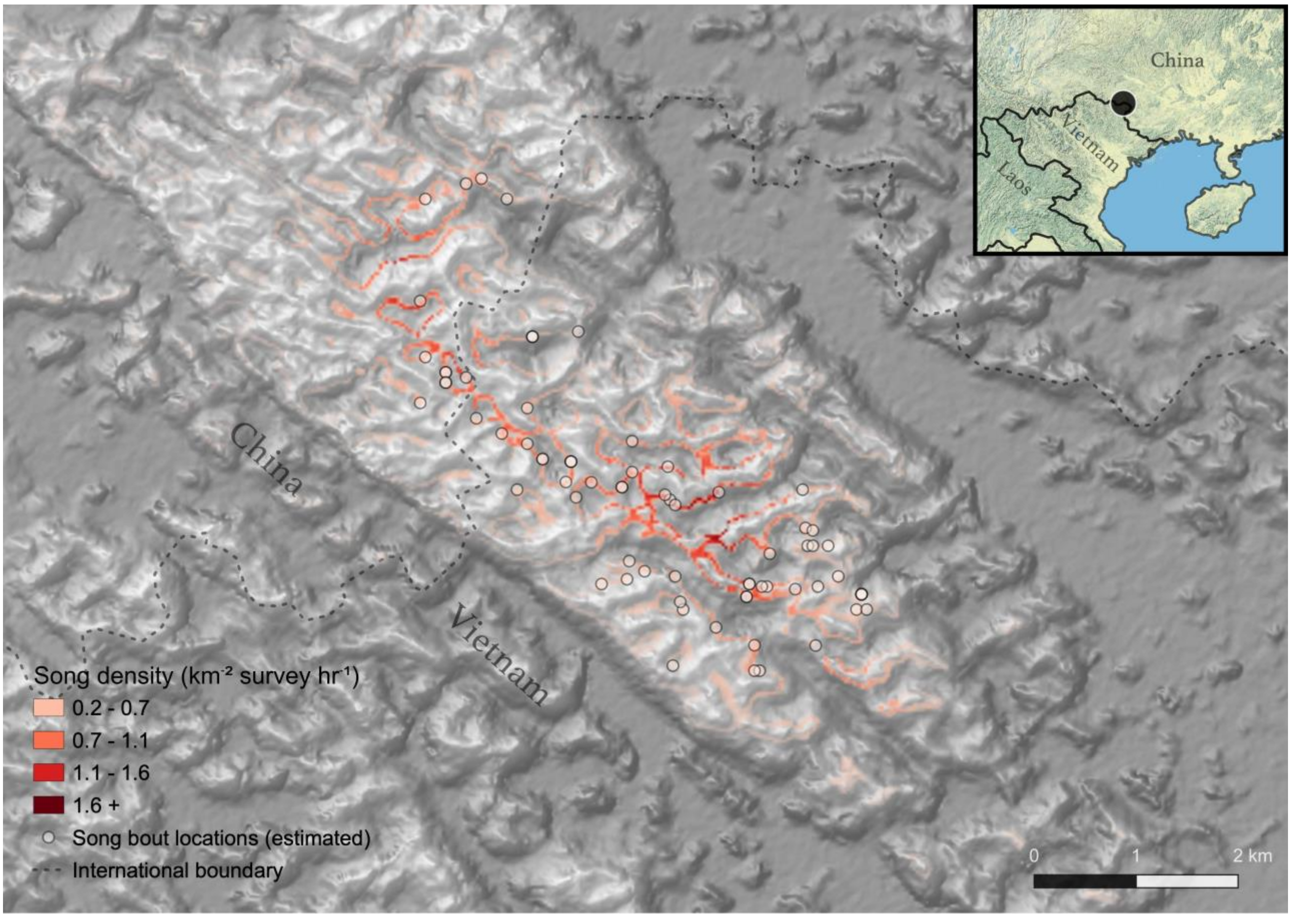

Supplement: Supplementary file 1 — Supplementary Information. [file 41598_2023_50838_MOESM1_ESM.pdf]
